# P-1384. Rates of Follow-Up Testing for *Chlamydia trachomatis* and *Neisseria gonorrhoeae* Among Active-Duty Service Members

**DOI:** 10.1093/ofid/ofae631.1560

**Published:** 2025-01-29

**Authors:** James J Marsh, David Manuel Aleman-Reyes, Joseph Marcus

**Affiliations:** Brooke Army Medical Center, San Antonio, Texas; San Antonio Uniformed Services Health Education Consortium, San Antonio, Texas; Brooke Army Medical Center, San Antonio, Texas

## Abstract

**Background:**

All patients who test positive for *Chlamydia trachomatis* (CT) and *Neisseria gonorrhoeae* (GC) should get retested at three months to evaluate for re-infection. The United States military has previously been reported to have high rates of CT and GC, but follow-up rates have not been established for active-duty service members (ADSM) who test positive for CT or GC. This study evaluates factors associated with follow-up CT or GC testing in ADSM.

**Figure:**
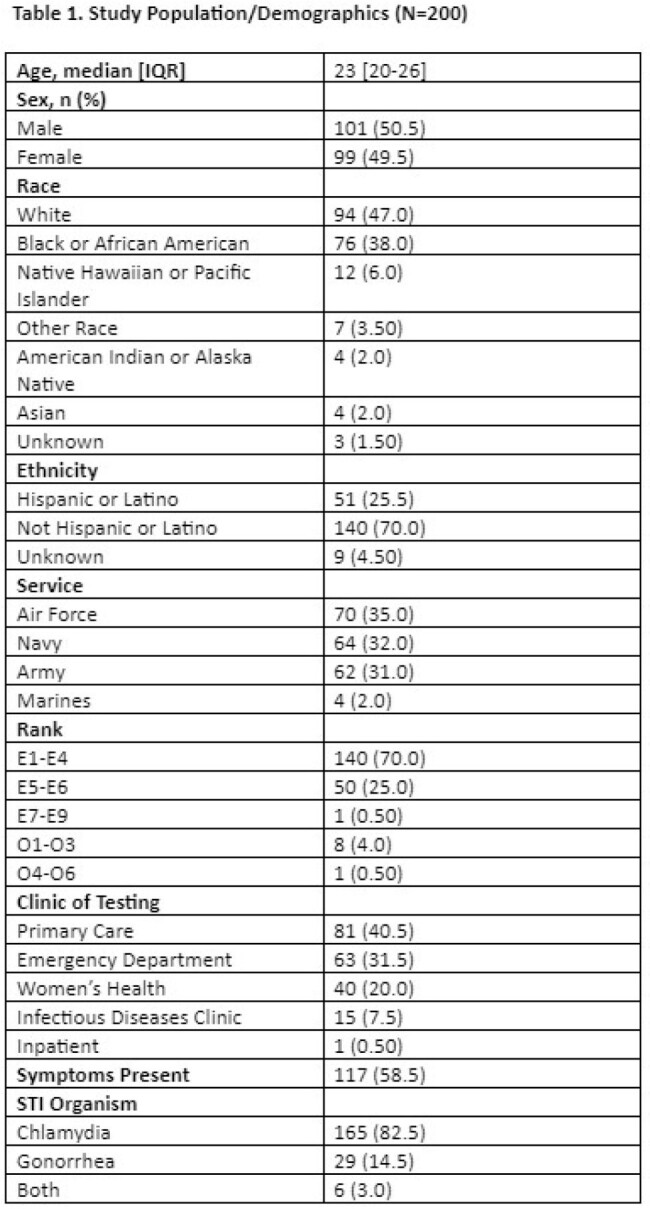

**Methods:**

Positive cases of CT and GC among ADSM who were stationed at Joint Base San Antonio between January and June 2023 were evaluated. Demographic characteristics, location of testing, and the presence of symptoms were described. A patient was determined to have had follow-up testing if they had a repeat CT/GC test within 3-12 months after their initial positive test. Follow-up rates were compared among groups based on demographics, clinical setting of testing, and presence of symptoms using chi-square or Fisher’s exact test.

**Figure:**
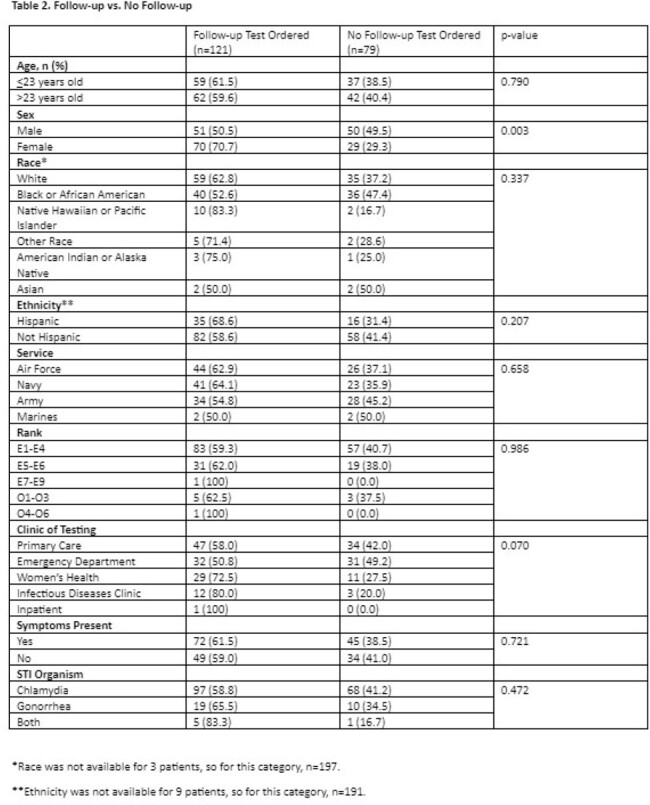

**Results:**

Of the 200 ADSM who tested positive for CT or GC during the study period, 101 (51%) were male and the median age was 23 [IQR: 20-26] (**Table 1)**. Women were significantly more likely to receive follow-up testing as compared to men (71% vs. 51%; p=0.003), without significant difference in follow-up testing by clinic, race, service, or original STI organism (**Table 2)**. Women who were symptomatic at time of initial test were significantly more likely than symptomatic men to have a follow-up test (76% vs. 49%; p=0.003), but this difference did not hold for asymptomatic patients (64% vs. 53%, p=0.28) (**Table 3**).

**Figure:**
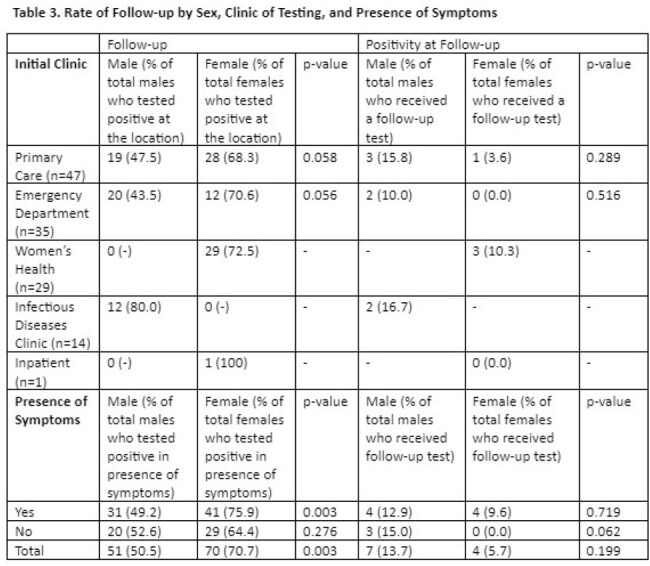

**Conclusion:**

Despite standardized follow-up testing recommendations, there were significantly lower follow-up testing rates in men, with the greatest difference in testing in patients who were symptomatic at presentation. While ADSM have universal access to care, the difference in follow-up testing between men and women based on presence of symptoms reinforces concerns about stigma in obtaining treatment and follow-up. Future work should study how standardization of guidelines in these populations promotes improvement in follow-up testing.

**Disclosures:**

**All Authors**: No reported disclosures

